# Editorial: Enzymes From Extreme Environments, Volume II

**DOI:** 10.3389/fbioe.2021.799426

**Published:** 2021-12-02

**Authors:** Noha M. Mesbah

**Affiliations:** Faculty of Pharmacy, Suez Canal University, Ismailia, Egypt

**Keywords:** extremozyme, extremophile, biotechnology, extreme enviroment, biocatalysis

The goal for a sustainable, biobased economy has thrust biotechnology and biocatalysis into the research spotlight. Biological catalysts are already employed in a myriad of applications. Polyethylene terephthalate hydrolases are used in biorecycling of plastic waste (Ellis et al., 2021). Amylases, proteases, lipases, mannanases and cellulases are ingredients in commercially available laundry detergents (Krüger et al., 2018). The textile industry utilizes recombinant cellulases for biological stonewashing of jeans, and catalases for biobleaching of fabrics (Kakkar and Wadhwa, 2021). Laccases and peroxidases are used in the bioremediation of environmental pollutants (Krüger et al., 2018). Lipases and esterases are catalysts in the synthesis of enantiopure pharmaceuticals and flavor and fragrance compounds by the pharmaceutical and cosmetic industries, and in the synthesis of agrochemicals such as herbicides (Jaeger and Eggert, 2002). Peptide-based drugs required for treatment of type II diabetes, multiple myeloma and multiple sclerosis require transferases for their production (Suresh et al., 2021). Most of the enzymes currently available do not tolerate harsh processing conditions and presence of additives and contaminants, and can lose activity due to extreme temperature, pH, ion concentrations and solvents.

Extremozymes perform the same catalytic functions as their non-extreme counterparts and are adapted to function under harsh industrial or environmental conditions with great versatility and robustness. Extremozymes are very attractive to biotechnological industries looking to replace non-biological catalysts with enzymes. The past 2 decades have seen great advancements in isolation and optimization of extremozymes, as well as development of enzymes with enhanced characteristics through protein-engineering and synthetic biology. Despite growing demand and extensive research however, the current enzyme market remains inadequate in meeting industrial demands. This Research Topic features some of the recent progress in extremozyme research, with a focus on applicability in industrial processes.

Oxidoreductases are one of the most abundant classes of enzymes in prokaryotic cells. Numerous redox-active molecules are present in cells, including amino acid residues, coenzymes (flavin dinucleotide, flavin mononucleotide), and metal ions and complexes such as heme and iron-sulfur clusters. Oxidoreductases use a wide range of electron donors and acceptors, including both organic and inorganic molecules. Oxidoreductases can also be used as biocatalysts for synthesis of fine molecules. The biotechnological potential and applications of extremophilic oxidoreductases were reviewed by Espina et al.. The authors focus on five major classes of oxidoreductases: laccases, hydrogenases, glutamate dehydrogenase, superoxide dismutase and catalase, and discuss their structural and functional features and current applications in industry, bioremediation and biofuel production.

Thermophilic enzymes are in high demand due to their stability at high temperatures in addition to resistance to denaturation and tolerance to solvents and high pressure. Bessonnet et al. describe identification of a thermostable nitrilase Nit_phym_ by heat-treatment of a collection 164 of heterologous nitrilases. Nit_phym_, from *Paraburkholderia phymatum*, has a half-life of 18 h at 60°C, broad pH range, and is resistant to numerous water-miscible organic solvents. Nit_phym_ is a promising candidate for large scale hydrolysis of nitriles as it was tolerant to up to 500 mM of substrate (Bessonnet et al.). Sadaqat et al. report on the cloning, purification and characterization of bifunctional Man/Cel5B with maximal activity at 85°C and pH 5.5 and activity against both cellulose and galactomannan (Sadaqat et al.). De Rose et al. describe functional and structural characterization of an extremely thermophilic, solvent resistant D-Lyxose isomerase TsLI with activity above 95°C. TsLI has very high specificity for D-lyxose. Structural characterization of TsLI revealed hallmark features of thermophilic proteins, including shorter surface loops, large amount of hydrophobic interactions, a large proportion of amino acid residues in α-helices and disulfide bridges (De Rose et al.).

Biological catalysis is critical for sustainable, environmentally friendly industry. Microbial extremozymes have been increasingly used over the past years for catalysis of reactions under difficult conditions commonly used in industries. Research highlighted in this Research Topic highlight latest progress in the field of extremozyme research and applicability of extremozymes in industry and bioremediation. The full potential of extremozymes has not been reached. Improvement in novel technologies such as function-based metagenomics, sequence-based metagenomics, single amplified genomes, systems biology, and protein-engineering will broaden the knowledge of extremozymes and facilitate development of tailored enzymes for specific processes, thereby bringing us closer to a sustainable future.
